# Efficacy and Safety of Tracnil™ Administration in Patients with Dermatological Manifestations of PCOS: An Open-Label Single-Arm Study

**DOI:** 10.1155/2020/7019126

**Published:** 2020-03-24

**Authors:** Ezhil Arasan Ramanan, Sailatha Ravi, K. R. Radha Anbu, Margaret Michael

**Affiliations:** ^1^Dr. VRE Research Laboratories, Chennai 600078, India; ^2^Medlish Communications, Chennai 600078, India; ^3^Anbu Clinic, Chennai 600020, India; ^4^Pristine Hospital and Research Centre, Bengaluru 560086, India

## Abstract

Myo-inositol's role in improving acne by reducing hyperandrogenism has been demonstrated in PCOS patients. Inositol and associated molecules display inhibitory properties against 5-*α* reductase, COX-2, and lipase enzymes in addition to their antimicrobial and anti-inflammatory properties. However, the role of myo-inositol is not well established in women patients with normal hormone levels but with clinical manifestations of PCOS. In this study, we evaluate the efficacy of Tracnil™, a combination of myo-inositol with folic acid and vitamin D3, in resolving acne in overweight women of menstruation age displaying normal hormone levels. It is a single-arm study conducted at 2 centers including 33 women with acne, hirsutism, and menstrual irregularities. Acne and hirsutism were assessed by manual lesion count, modified Cook's scale, and modified Ferriman–Gallwey hirsutism score (mFGHS). Hormone levels and safety parameters were assessed throughout the study. Our results show that Tracnil™ monotherapy could drastically reduce acne-related lesions of both inflammatory and noninflammatory types as quickly as 8 weeks. Additionally, it improves hirsutism and menstrual irregularities. Adverse reactions were negligible during the whole study period with no drastic side effects reflected by a modulatory effect on hormone levels. Despite the subjects having normal hormone levels, the acne treatment with myo-inositol and vitamin D3 shows improvement in hirsutism and regularization of menstrual cycle. Therefore, we attribute the mechanism of action of Tracnil™ to modulation of receptor sensitivity to sex hormones or other downstream processing events. Tracnil™ may be considered as a first-line treatment for dermatological manifestations of PCOS even in the absence of significant hormonal abnormalities. This treatment is practically implementable in a dermatologists's office practise.

## 1. Introduction

Acne vulgaris is a disease of the pilosebaceous unit, characterized by release of inflammatory mediators, hyperkeratinization, increased sebum production, and *Cutibacterium acnes* colonization [1]. Hypersensitivity of the glands to normal circulating androgen levels is common in acne [2]. Persistence of acne in about 12% of women beyond teenage may reflect underlying systemic diseases or syndromes [3, 4]. The acne lesions in mild-to-moderate cases could vary from noninflammatory comedones to inflammatory papules and pustules.

Acne, hirsutism, alopecia, and acanthosis nigricans are some of the common dermatological manifestations in women with polycystic ovarian syndrome (PCOS) [5, 6]. Management of PCOS warrants a complex multidisciplinary approach involving endocrinologists, gynecologists, and dermatologists. Although hormonal therapy does not ensure any permanent correction of the underlying pathology, it is often employed [2]. The complicated process of eliminating contraindications including weight gain, thromboembolic disorders, and cardiovascular diseases favors safer nonhormonal therapies.

A newer nonhormonal alternative for PCOS is myo-inositol, a common stereoisomer of inositol, abundant in plants and animal tissues [7, 8]. Myo-inositol administration has been widely shown to modulate hormonal fluctuations and improve fertility in patients with PCOS, by correcting insulin resistance [9–11]. Inositol helps improve skin condition by reducing hyperandrogenism [12] or many other closely related mechanisms of action. Myo-inositol hexaphosphate (phytic acid) displays antimicrobial and anti-inflammatory properties and is a potent inhibitor of 5-*α* reductase, COX-2, and lipase enzymes [13–18]. This study shows that a proprietary combination of myo-inositol with folic acid and vitamin D3 (as Tracnil™) is highly effective in resolving acne in overweight women of menstruation age displaying normal hormone levels. Our study also addressed the menstrual irregularities and other dermatological manifestations that possibly suggest an underlying PCOS.

## 2. Subjects and Methods

### 2.1. Open-Ended Single-Arm Study

**Figure 1:**
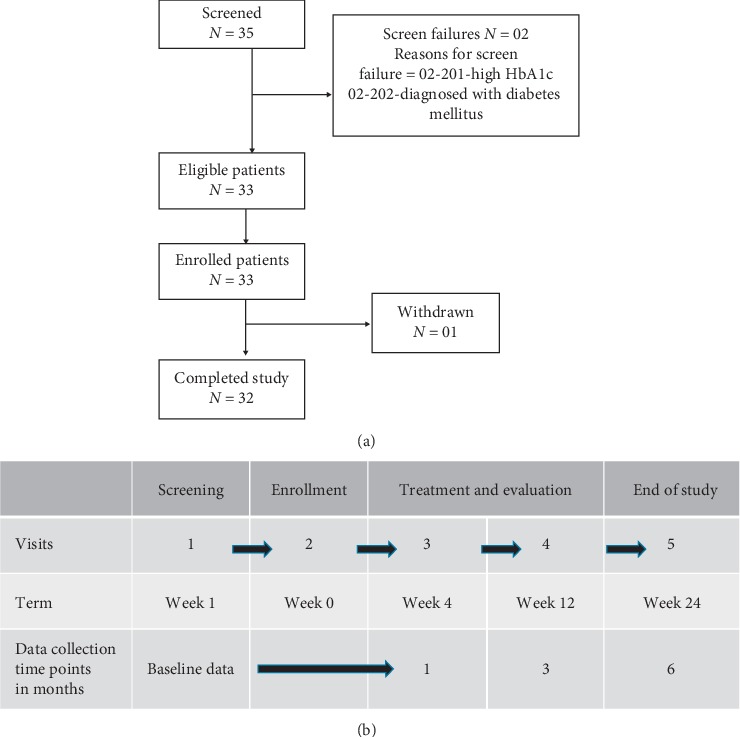
Schematic representation of the study design: (a) consort flow diagram and (b) sequence of events and time line of the study.

The study was conducted in compliance with ICH-GCP at two dermatology centers involving about 33 eligible female patients. The study was conducted from 20 March 2018 to 09 November 2018. An overview of the study design is given in Figure 1.

### 2.2. Sample Size Requirement

For obtaining a 60% reduction in acne over a period of 6 months (baseline lesion count of mean ± SD of 14 ± 1.7), with 95% confidence interval and 80% power, we estimated that a sample size of 28 participants was required to conduct the study. Considering a drop out ratio of 10%, it was deduced that we require 31 participants at the time of screening. Each participant provided an informed consent at the time of enrollment. The study protocol id, CHC_TRA_HA01_17 had been carried out in accordance with the ethical principles of the declaration of India and approved by the ethics committee of Rangammal Hospital, India. The study was registered with CTRI (Clinical Trials Registry of India) number CTRI/2018/03/012481 http://ctri.nic.in/Clinicaltrials/showallp.php?mid1=22895&EncHid=&userName=tracnil.

Females 18 to 45 years of age, with a BMI ≥ 28, were included. All patients had mild-to-moderate acne of about 5–50 noninflammatory lesions (closed comedo or degree 1 open comedo) and/or inflammatory facial lesions (papule and degree 2 pustule), except in the nose region. Most patients had hirsutism with a modified Ferriman–Gallwey hirsutism score (mFGHS) of 8 to 20.

Patients posing certain risk for the treatment or having other condition causing ovulatory disorders, androgen excess, or already taking hormonal treatment or with a positive result for urinary hCG or gestation period or with diabetes mellitus (HbA1C > 6.5) were excluded.

All patients received sufficient quantity of 5 g sachets of Tracnil™ (containing myo-inositol 2000 mg; folic acid 1 mg and vitamin D3 1000 IU) dissolved in water, twice a day, before meals (breakfast and dinner), for a period of 6 months (24 weeks).

At the time of screening, patient demographics (Table 1), medical, and menstrual history were collected. Vital signs, hematology, and biochemical parameters were recorded, and routine urine analysis and safety assessments were carried out at the time of screening and during the study at each visit.

Primarily, percentage reduction of total acne lesion on the face in comparison with the baseline lesion counts and changes in serum hormone levels during follicular phase ((luteinizing hormone [LH], follicle-stimulating hormone [FSH], testosterone (total and free), androstenedione, dehydroepiandrosterone sulfate, prolactin, insulin and homeostatic model assessment [HOMA] index) were estimated.

Secondary parameters which were evaluated were changes in menstrual cycles, Global Acne Assessment (GSA), Acne Quality of Life Index (AQOLI), and hirsutism score of mFGHS. All the adverse events were coded using MedDRA version 16.0.

### 2.3. Statistical Analysis

The data entry was performed and validated using the double data entry interactive method. Data collected at baseline was compared with that at the end of the study. The laboratory investigation reports were reviewed for its clinical significance in case of any abnormalities. Statistical analysis was performed using student's paired *t*-test for means on the data set, and the error bars are represented as standard error mean.

## 3. Results

In this single-arm study, efficacy and safety of oral Tracnil™ were assessed in thirty-three female patients with associated symptoms of mild-to-moderate acne and hirsutism.

**Figure 2:**
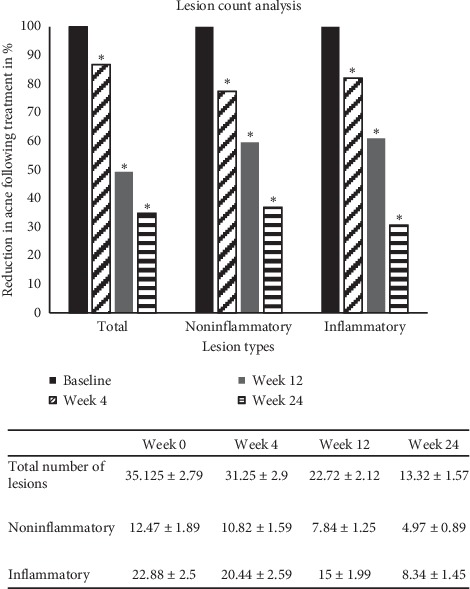
Quantitative analysis indicating percentage reduction in acne lesions. Lesion counts of total acne including the inflammatory and the noninflammatory types at week 0 (100%), 4, 12, and 24 are represented. ^*∗*^Statistical significance of *P* < 0.05, based on Student's *t*-test. The table provides the corresponding values as average ± SEM.

Demographic information (Table 1) was collected. The enrolled subjects had a mean BMI of 29.1 ± 2.05 (mean ± SD) with presentation of acne in the face and neck areas (Figure 2) and also had an irregular menstrual cycle. Their urine pregnancy test was confirmed to be negative, and their hormone levels which were tested were found to be normal. Hematology, other biochemical parameters, and the HOMA index for insulin resistance did not show any significant abnormality (Table 2). Following administration of Tracnil™ sachets, the study results were monitored on week 4, week 12, and week 24.

### 3.1. Impact on Acne-Associated Lesions

**Figure 3:**
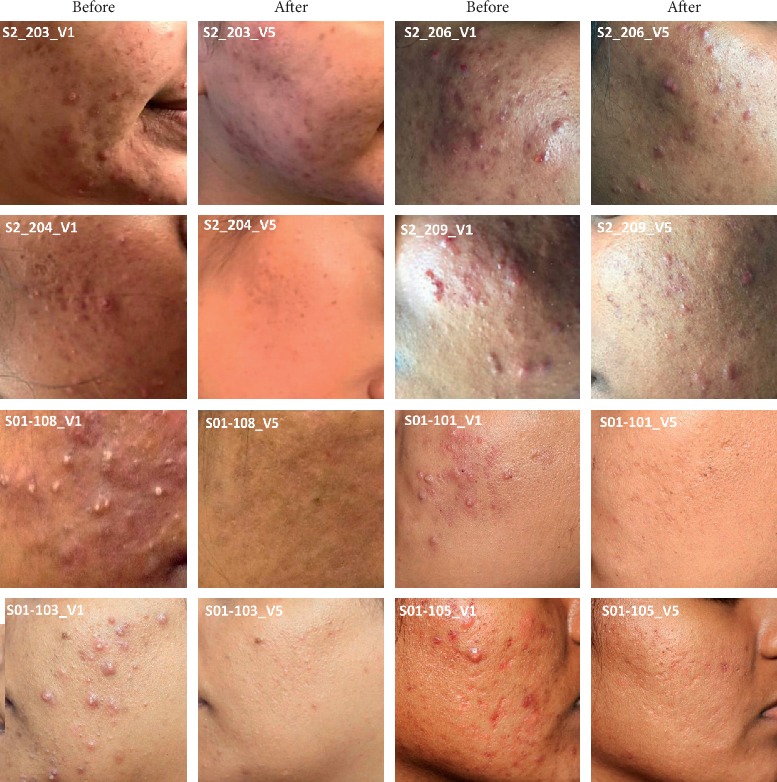
Representative skin lesion images before (week 0) and after (week 24) treatment. The different labels on the picture represent different patient ids including the site number (S1 or S2), patient number (###), and visit (V1 or V5).

The efficacy of Tracnil™, mainly containing myo-inositol, in acne treatment was evaluated by manual lesion count analysis. Results (Figure 2) indicate that compared to the baseline, by week 4, 12, and 24, there was a significantly consistent decreasing trend in the acne-associated lesions. A significant reduction (*P* < 0.01) in the total lesions including the inflammatory and the noninflammatory types was observed in a very short time of week 4. There was almost 50% reduction in the acne-associated lesions by week 12. Photographic evidence (Figure 3) indicates a visible reduction in acne-associated lesions following Tracnil™ administration.

**Figure 4:**
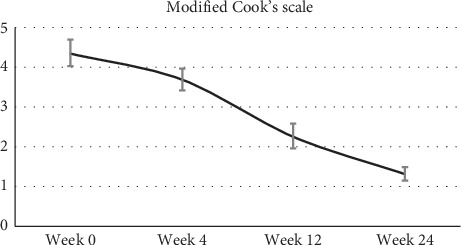
Global acne assessment using modified Cook's scale: The data represent the modified Cook's scale analysis made during each visit (at week 9, week 4, week 12, and week 24). The error bars are average ± SEM.

Score from the Investigator Global Acne Assessment using modified Cook's scale at week 0, week 4, week 12, and week 24 was summarized using descriptive statistics. From a mean baseline score of 4.34 ± 0.33, the score decreased significantly with further visits to a mean value of 1.3 ± 0.17 by week 24 (Figure 4).

### 3.2. Impact on Hirsutism and Alopecia

**Figure 5:**
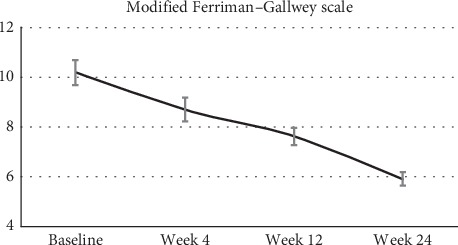
Modified Ferriman–Gallwey scale for scoring hirsutism (mFGHS). The data represent the mFGHS analysis made during each visit (baseline or week 0, week 4, week 12, and week 24). The error bars are average ± SEM.

Results from mFGHS indicate a considerable improvement in hirsutism during each visit. Compared to the baseline with a mean score of 10, the mFGHS reduced to 8.6 by week 4 to 7.4 by week 12 and to 5.8 by week 24 (Figure 5).

Subjectively, about 80% of the women at the time of enrollment complained about hair loss. By the end of the study period, only 20% of the women had lingering hair loss issues.

### 3.3. Improvement in Menstrual Cyclicity

**Figure 6:**
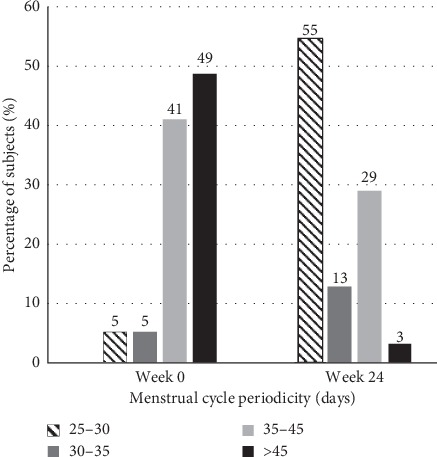
Distribution of menstrual cycle variation among the subjects during the study period. The different bar patterns represent the menstrual cycle distribution in days among the patients having 25–30-day, 30–35-day 35–45-day, or >45-day cycle, respectively. The enrolled patients displayed normal hormonal levels.

Menstrual data pertaining to frequency, duration, and flow was collected throughout the study. At week 0, 10% of the patients reported having a normal menstrual cycle (25–35 days). Following a 24-week treatment with myo-inositol, the proportion of patients with the normal cycle improved drastically to 68% (Figure 6). Moreover, these results correspond with a significant reduction to 3% of the patients with >45-day menstrual cycle compared to 49% at the time of enrollment.

**Figure 7:**
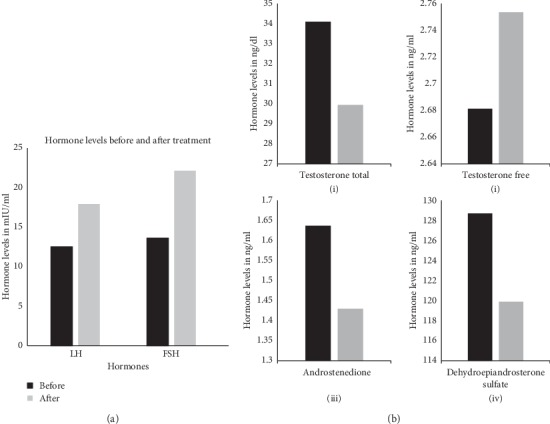
Variation in hormone levels before and after treatment with Tracnil™. The different hormone levels at week 0 (black bar) and at week 24 (grey bar) are presented as average ± SEM. (a) analyzes LH and FSH levels. (b) compares testosterone total and free levels (i and ii) and androstenedione and dehydroepiandrosterone sulfate levels (iii and iv). Statistical analysis using Student's *t*-test (*P* > 0.05) was used to assess any significant changes in levels.

Hormone levels of the patients evaluated for LH, FSH, and the ratio of LH to FSH measured on week 0 and on week 24 did not indicate any significant change (Figure 7(a)). Similarly, assessment for testosterone (free and total), androstenedione, and dehydroepiandrosterone sulfate conducted on week 0 and on week 24 did not show any significant change (Figure 7(b)). Moreover, the levels were within the normal range (Tables 3–5). Also, assessment for prolactin, insulin fasting serum, and glucose fasting serum did not show any significant marked changes (Table 5).

### 3.4. Psychological Assessment of Patients Based on Their Aesthetics

**Figure 8:**
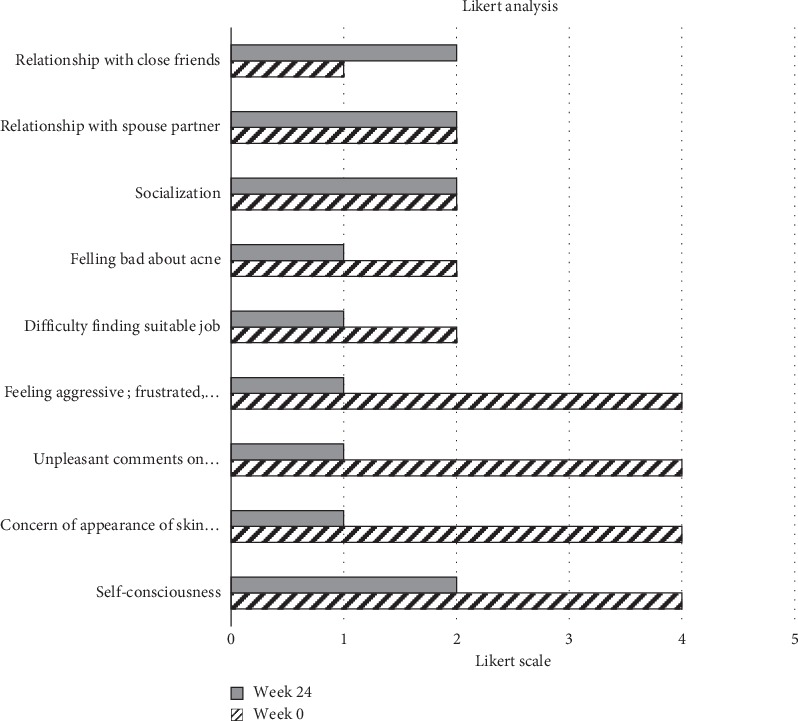
Likert scale analysis (scale of 0–5) for psychological assessment of patients with acne following treatment. The assessment parameters are listed on the left, and the bars correspond to the response at week 0 or at week 24.

Psychological assessment of patients [14] using the Likert scale was done at weeks 4, 12, and 24. A total of 11 parameters were assessed to measure the attitude of the respondents towards acne. Patients indicated their level of agreement in a questionnaire, with statements related to stimulus objects ranging over 5 grades. The qualitative analysis indicates that self-consciousness, social interactions, and relationship with immediate friends and family improved by the end of the study (Figure 8).

### 3.5. Safety of Tracnil™ Administration

Most of the adverse events reported were mild in nature, and there were no serious adverse events observed during the study. Most common AE were minor gastrointestinal disturbances, such as gastritis and vomiting.

Discussion: this study evaluates the efficacy of a proprietary combination of myo-inositol with folic acid and vitamin D3 (Tracnil™) as a first line of treatment option for acne, hirsutism, and menstrual irregularity in overweight or obese women. According to NIH 1990 criteria, clinical and biochemical hyperandrogenism along with oligo/amenorrhea anovulation [19] serve as factors in the diagnosis of PCOS [20]. Rotterdam 2003 criteria defined PCOS to be associated with any two of the symptoms including acne, hirsutism, androgenic alopecia, hormonal fluctuations, and insulin resistance that further needed verification of morphology using an ultrasound diagnosis [21].

Clinically, acne, hirsutism, androgenic alopecia, and menstrual cycle irregularities are manifestations of hyperandrogenism and PCOS [5]. A clinical study diagnosed that out of 950 patients having hyperandrogenism, 72.1% had PCOS [22, 23]. In another study on hyperandrogenic patients, 78.4% of hirsute subjects had PCOS [20].

In a real-world setting, when an acne patient approaches with other clinical manifestations of PCOS, the dermatologist's clinical dilemma is on investigating further for a complete hormonal and ultrasound profile. The need to ascertain nonexistence of contraindications for oral contraceptives and balancing core hormonal therapies add complexity that demands a cross-specialty teamwork.

In general, nonhormonal treatments with inherently lower side effects and shorter duration of treatment are preferred over hormonal therapies. On the contrary, the topical treatments do not address other manifestations of a possible PCOS [24]. Lifestyle changes are essential in any context. Here, we propose a nonhormonal therapy comprisingmyo-inositol, folic acid, and vitamin D3 , as a first-line treatment alternative for overweight women with acne, hirsutism, and menstrual irregularity.

The treatment had 3 main impacts on the enrolled patients.

Firstly, Tracnil™ administration showed a 69.3% reduction (*P* < 0.01) in inflammatory acne lesions and 63% reduction of noninflammatory lesions (*P* < 0.01) (Figure 2). Considering that Tracnil was the only stand-alone treatment given to these tough-to-treat acne patients, it offers an extremely promising therapeutic value. Moreover, an increasing trend in the AQOLI assessment and the Likert scale analysis suggests a profound impact on the psychological state of most patients.

Secondly, Tracnil administration has been extremely effective in treating hirsutism in that the mean baseline hirsutism score of 10 dropped to 6 (Figure 5). An mFGHS score of >8 is normally considered to ascertain hirsutism in female populations [25, 26]. The prevalence and association of hirsutism was shown by Wijeyaratne et al., earlier to be more severe in Indian populations [27]. Our results showed for the first time a promising treatment option for hirsute Indian women.

The third therapeutic value of Tracnil™ is on menstrual irregularities. While at the time of enrollment, menstrual irregularity was observed in 70% of the subjects, and it markedly dropped to 3% by the end of the study period. Regidor et al. has observed similar results with myo-inositol and folic acid administration on German women [11]. A nonhormonal preparation with such a potential to regularize menstrual cycle in these women is of great significance.

In accordance with other previous studies [24, 28], there are no associated adverse events reported during the study making Tracnil™ a more appealing nonhormonal treatment option for women presenting acne with other clinical manifestations of PCOS.

In a double-blinded placebo-controlled study, myo-inositol administration could positively impact metabolic and hormonal parameters. This was accompanied by a significant decrease of the total and free testosterone serum levels that subsequently lead to an improvement of skin problems such as acne or hirsutism [29]. Previous studies have evidenced a vitamin D-mediated response (supplemented at 1000 IU/day) resulting in up to 30% reduction in acne [30, 31]. Since in our study we see a drastic 50% reduction by week 12, it appears to be a potential for a valuable additive effect between myo-inositol and vitamin D.

Although acne in women with clinical manifestations of PCOS is associated with androgen fluctuations, there are instances of normal androgen level in some cases of acne vulgaris and increased receptor sensitivity to circulating androgens [5, 32, 33]. Schmidt et al. suggested that androgen action at the target organ level in dermatoses might be independent of peripheral serum levels of hormones [34]. Androgen and androgen receptors play important yet distinct roles in skin-related disorders. A study conducted on the androgen receptor knockout mice model [35] and a follow-up study by Lai et al. [36] discuss that androgen receptors could be a better target than the androgen itself in treating skin diseases. In our trial study, since we do not find associations with hormonal fluctuations, mechanistically, we attribute the myo-inositol activity to be due to modulation of androgen receptor sensitivity [37]. Alternatively, there might have been localized fluctuations in androgen levels that go undetected.

Myo-inositol derivative has been known to have an inhibitory action against 5-*α* reductase activity, thereby inhibiting the sensitization of receptors to androgen levels [2, 16, 38]. This could also possibly explain the benefits derived out of myo-inositol, folic acid, and vitamin D formulations' clinical value in controlling acne [39, 40].

Our findings that Tracnil™ could drastically reduce acne-related lesions of both inflammatory and noninflammatory types as quickly as 4 to 8 weeks in patients possessing clinical manifestations of a possible PCOD has important clinical implications. The study did not explore the possible additive value of topical agents in addition to Tracnil, but it appears that there is a great potential for a substantial benefit in controlling tough-to-treat acne. Despite the subjects having normal hormone levels, the acne lesions succumb to the myo-inositol treatment along with improvement in hirsutism and regularization of the menstrual cycle. Therefore, we attribute the mechanism of action of Tracnil™ to modulate the sensitization of the receptors to hormones or other downstream processing events, or possibly by inhibition of 5-*α* reductase activity. Our study poses limitations owing to its small sample size and absence of the placebo control group. Further, a thorough investigation of hormone levels and the associated enzyme activity needs to be pursued for a complete understanding of the mechanistic details. However, the results are quite promising that a large-scale randomized control trial could be conducted.

Through this study's findings, Tracnil™ has demonstrated clinical benefits even in patients without any manifested biochemical or hormonal imbalance in accordance with other studies. Overall, Tracnil™ administration may be of great significance as a valuable newer therapeutic intervention easily available to the physicians for satisfactory dermatological outcomes with greatly improved quality of life of the patients.

## Figures and Tables

**Table 1:** Patient demographics collected on enrollment.

Demographic characteristics	
Gender	
Female	

Number of subjects	
*n* (%)	33 (100)

Age in years	
Mean (SD)	26.4 (6.62)
Minimum	20
Median	24.0
Maximum	44

Race, *n* (%)	
Indian	33 (100)

Height in cm	
Mean (SD)	153.3 (7.10)
Minimum	135
Median	153
Maximum	166

Weight in kg	
Mean (SD)	68.5 (6.74)
Minimum	53
Median	69
Maximum	85

BMI in kg/m^2^	
Mean (SD)	29.1 (2.05)
Minimum	25
Median	29.1
Maximum	33.6

**Table 2:** Biochemical parameters measured during the course of study.

Parameter	Units	Ref range	Week 0	Week 4	Week 12	Week 24
			Average value ± SEM			
Blood glucose	mg/ml	90–120	121.4 ± 5.12	113.66 ± 2.41	113.26 ± 3.16	111.72 ± 3.70
Albumin	g/dl	3.2–4.8	4.36 ± 0.046	4.37 ± 0.054	4.31 ± 0.061	4.35 ± 0.046
Alkaline phosphatase	U/l	42–98	90.33 ± 3.70	85.59 ± 3.37	85.50 ± 3.34	90.60 ± 3.93
ALT	U/l	10–28	16.52 ± 0.96	17.11 ± 1.42	17.2 ± 1.48	21.11 ± 3.2
AST	U/l	<31	22.02 ± 1.07	22.54 ± 1.37	22.43 ± 1.27	27.11 ± 2.44
Bilirubin (direct)	mg/dl	<0.3	0.17 ± 0.015	0.16 ± 0.012	0.17 ± 0.016	0.18 ± 0.013
Bilirubin (indirect)	mg/dl	0–0.9	0.39 ± 0.023	0.37 ± 0.025	0.41 ± 0.033	0.43 ± 0.033
Blood urea nitrogen	mg/dl	7–25	9.06 ± 0.58	10.47 ± 0.52	10.51 ± 0.62	11.71 ± 0.65
Glomerular filtration rate	ml/min/1.73 m^2^	≥90	120.17 ± 1.75	122.66 ± 2.12	122.06 ± 2.80	123.13 ± 2.09
Serum calcium	mg/dl	8.8–10.6	9.24 ± 0.077	9.35 ± 0.11	9.23 ± 0.12	9.51 ± 0.064
Serum creatinine	mg/dl	0.5–0.8	0.65 ± 0.014	0.63 ± 0.016	0.64 ± 0.024	0.62 ± 0.015
HOMA index value	—	0.7–2	1.44 ± 0.15	—	—	1.88 ± 0.19

**Table 3:** The endocrine and metabolic assessment of patients before (week 0) and after (week 24) the treatment with Tracnil™.

Group	LH^*∗*^ (mIU/ml)	FSH^*∗*^ (mIU/ml)	LH/FSH^*∗*^ ratio
Before	12.51 ± 2.29	13.61 ± 4.84	1.692 ± 0.223
After	17.91 ± 3.17	22.13 ± 5.73	1.393 ± 0.217

Data shown as mean ± SEM for a sample size of *N* = 32 patients. ^*∗*^Student's *t*-test indicated no difference (*P* > 0.05) between the levels before and after treatment.

**Table 4:** The endocrine and metabolic assessment of patients before and after the treatment with Tracnil™.

Group	Testosterone total^*∗*^ (ng/dl)	Testosterone free^*∗*^ (pg/ml)	Androstenedione^*∗*^ (ng/ml)
Before	34.10 ± 2.83	2.681 ± 0.264	1.637 ± 0.163
After	29.94 ± 2.91	2.754 ± 0.229	1.43 ± 0.178

Data shown as mean ± SEM for a sample size of *N* = 32 patients. ^*∗*^Student's *t*-test indicated no difference (*P* > 0.05) between the levels of each group before and after treatment.

**Table 5:** The endocrine and metabolic assessment of patients before and after administration of Tracnil™.

Group	Dehydroepiandrosterone sulfate^*∗*^ (*μ*g/dl)	Prolactin^*∗*^ (ng/ml)	Insulin fasting serum^*∗*^ (*μ*U/ml)	Glucose fasting serum^*∗∗*^ (mg/dl)
Before	128.7 ± 13.8	11.91 ± 1.27	7.048 ± 0.860	91.44 ± 2.14
After	119.9 ± 14.1	9.82 ± 1	9.58 ± 1.78	82.32 ± 3.04

Data shown as mean ± SEM for a sample size of *N* = 32 patients. ^*∗*^Student's *t*-test indicated no difference (*P* > 0.05) between the levels of each group before and after treatment. ^*∗∗*^Student's *t*-test indicated significant difference (*P* < 0.05) in the levels of each group before and after treatment.

## Data Availability

Readers can access the data underlying the findings of this study by contacting the author through e-mail at drpharmaindia@gmail.com.
